# SRSF9-Mediated Exon Recognition Promotes Exon 2 Inclusion in *Mecp2* Pre-mRNA Alternative Splicing

**DOI:** 10.3390/ijms26073319

**Published:** 2025-04-02

**Authors:** Saya Oshizuki, So Masaki, Satoshi Tanaka, Naoyuki Kataoka

**Affiliations:** Laboratory of Cellular Biochemistry, Department of Animal Resource Sciences, Graduate School of Agricultural and Life Sciences, The University of Tokyo, Tokyo 113-8657, Japan

**Keywords:** MeCP2, alternative splicing, exon recognition, SRSF9

## Abstract

Alternative splicing is one of the processes that contributes to producing a vast protein diversity from the limited number of protein-coding genes in higher eukaryotes. The *Methyl CpG Binding Protein 2* (*Mecp2*) gene, whose mutations cause Rett syndrome, generates two protein isoforms, MeCP2E1 and MeCP2E2, by alternative splicing. These isoforms likely possess non-redundant functions. However, the molecular mechanism for *Mecp2* pre-mRNA alternative splicing remains to be understood. Here, we analyzed the alternative splicing mechanism of MeCP2 pre-mRNA and found that exon 2 is efficiently recognized through adjacent strong splice sites. In addition, exonic splicing enhancer (ESE) in exon 2 plays an important role in exon 2 inclusion, which is highly likely to be mediated by SRSF9.

## 1. Introduction

Pre-mRNA splicing is the process of removing introns and ligating exons to produce mRNAs [1]. Since most nuclear-encoded protein-coding genes are separated by introns in higher eukaryotes, pre-mRNA splicing is essential for gene expression. Alternative splicing is one of the pre-mRNA splicing modes which yields a vast protein diversity from the limited numbers of protein-coding genes in higher eukaryotes [2,3,4,5]. Alternative splicing utilizes alternative exons and produces many different species of mRNAs from a single precursor of mRNA (pre-mRNA). Both cis-acting and trans-acting factors are involved in alternative splicing. In higher eukaryotes, it has been assumed that exon recognition is a predominant mechanism in splicing [2,6]. In this system, exon is recognized as a unit through the interaction between U2 snRNP on the upstream 3′ splice site region and the U1 snRNP downstream 5′ splice site [2,6]. This bridging is occasionally enhanced by an exonic splicing enhancer (ESE). The major ESE binding proteins are Serine–Arginine-rich splicing factors (SRSFs) [7,8]. SRSFs bind to ESE through their RNA binding domain and interact with both U2AFs and the U170K protein via their Arginine–Serine-rich (RS) domain, resulting in the promotion of exon recognition [2,6].

*Methyl-CpG-Binding Protein 2* (*Mecp2*) is one of the genes that produces alternative splicing isoforms. MeCP2 was first identified biochemically by its binding activity to methylated DNA [9,10]. MeCP2 is a transcription regulator that binds to methylated cytosines in promoter regions to cause both the activation and inhibition of downstream target genes [9,10]. Later, *MeCP2* was found to be a gene responsible for Rett syndrome [11]. It turned out that there was another protein isoform termed the MeCP2E1 protein [12,13,14,15], which renamed the originally identified protein isoform to the MeCP2E2 protein [16]. These two protein isoforms are translated from *Mecp2e1* (e1) mRNA and *Mecp2e2* (e2) mRNA, which are produced by exon 2 exclusion (for e1) and inclusion (for e2) from *Mecp2* pre-mRNA, respectively. The e1 mRNA encodes the MeCP2E1 protein, which is the major protein isoform in the brain [12,13,14,15], whereas the e2 mRNA encodes the MeCP2E2 protein isoform [16]. These two protein isoforms share functional domains but have different amino-terminal sequences [12,13]. In previous studies, the expression patterns of both splicing isoforms were reported [12,13]. While one study showed that most of the human tissues they tested displayed a *MeCP2e1*-dominant pattern [12], the other reported that most human tissues, except the brain, mainly expressed a *MeCP2e2* isoform [13]. The functional differences between two splicing isoforms have been investigated. Transgenic mice expressing either MeCP2e1 or MeCP2e2 cDNAs in brain cells were generated and crossed with mice lacking *Mecp2*. The ability of each of the variants to compensate for the lack of the endogenous *MeCP2* gene was determined. It was observed that expression of either of the isoforms mitigated the phenotypic consequences from the lack of *Mecp2* and allowed for *Mecp2*^−/Y^ mice to survive to adulthood, though the survival rate of *MeCP2e1* was higher than that of *e2* [17]. This demonstrates that both isoforms have redundant and significantly overlapped roles in mice. However, other studies revealed that knockout of the MeCP2E1 protein exhibited Rett syndrome-like phenotype in mice [18], whereas MeCP2E2-specific knockout displayed no Rett syndrome-like phenotype, but did show placenta development deficiency [19]. These two isoforms exhibit differences in expression patterns, and furthermore, they have different DNA binding affinities and different binding proteins [12,13,20]. These results strongly suggest that two protein isoforms of MeCP2 harbor their non-redundant functions in biological processes in mice and humans. However, mechanisms for MeCP2 alternative splicing remain to be elucidated.

In this study, we tried to uncover the mechanism of MeCP2 alternative splicing by taking advantage of mini-gene splicing reporters. Our results indicate that the exon recognition step for exon 2 is critical for e2-type splicing, which is mediated by the strong splice sites adjacent to exon 2 and ESE within exon 2 bound to SRSF9.

## 2. Results

### 2.1. Establishment of an Alternative Splicing Reporter for Mecp2 Pre-mRNA

**Figure 1:**
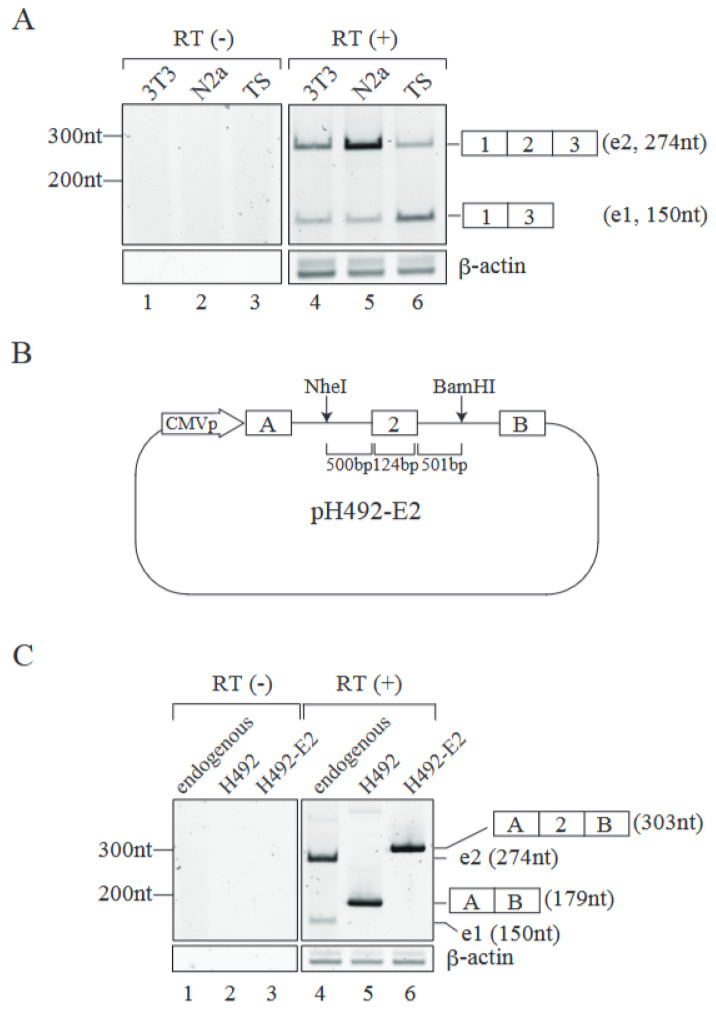
A mini-gene reporter with Mecp2 exon2 reflects the endogenous Mecp2 alternative splicing pattern in mouse Neuro 2a cells. (**A**) RT-PCR analyses for Mecp2 alternative splicing patterns with total RNAs from three different mouse cells (upper panel). Schematic representations of RT-PCR products are shown at right side with their length in nucleotides. The b-actin mRNA was used as an internal control (lower panel). The main replicon was an expected size product of β-actin mRNA, while the larger product could be a PCR-artifact. RT-PCR reactions without reverse transcriptase (RT (−)) were carried out for both Mecp2 and b-actin as negative controls. N2a, Neuro 2a cell; 3T3, NIH3T3 cell; TS, mouse Trophoblast Stem Cell. Representative results from three independent experiments are shown. (**B**) A schematic representation of the mini-gene splicing reporter plasmid that includes exon 2 of the mouse Mecp2 gene, pH492-E2. This plasmid was transfected into mouse Neuro 2a cells and the pre-mRNAs were transcribed from the cytomegalovirus (CMV) promoter (CMVp). Letters A and B in the boxes represent exon A and B, respectively, which are originally included in the H492 vector. (**C**) The RT-PCR products derived from the reporter mRNAs recovered from transfected mouse Neuro 2a cells were visualized on acrylamide gel (lanes 5 and 6 of the right upper panel). Splicing patterns of endogenous Mecp2 mRNA are also shown (lane 4 of the upper right panel). Schematic representations of RT-PCR products are indicated on the right side with their nucleotide length. The PCR products derived from endogenous e1-type and e2-type mRNAs are indicated as e1 and e2 with their length in nucleotides, respectively. The b-actin mRNA was used as a control (lower right panel). As negative controls, RT-PCR reactions without reverse transcriptase (RT (−)) were performed (lanes 1–3, left panels). Representative results from three independent experiments are shown.

To uncover the *Mecp2* pre-mRNA alternative splicing mechanism, we first determined endogenous *Mecp2* splicing patterns in three mouse cell lines. RT-PCR analyses from those cell lines demonstrated that NIH3T3 and Neuro 2a cells had more of the e2-type (exon 2 included) product than that of e1 (exon 2 excluded) (Figure 1A lanes 4 and 5), whereas mouse Trophoblast Stem Cells (TS), which can be differentiated into placenta cells in vitro [21], showed a dominant pattern of e1-type product (Figure 1A, lane 6). These results indicate that MeCP2 pre-mRNA undergoes alternative splicing in a cell-type-specific manner.

To analyze the molecular mechanism of MeCP2 alternative splicing, we prepared a splicing reporter based on the H492 vector that contains a portion of the mouse *Mecp2* gene including exon 2 and approximately 500 bp of the flanking introns at both sides (Figure 1B). This plasmid, termed pH492-E2 reporter, was introduced into mouse Neuro 2a cells, and total RNAs were extracted from the transfected cells. RT-PCR analyses revealed that pre-mRNA from the pH492-E2 reporter was subjected to alternative splicing and almost e2-type mRNAs were produced (Figure 1C, lane 6). This pattern was similar to that of endogenous *Mecp2* alternative splicing in Neuro 2a cells, in which the e2 type is dominant (Figure 1C, lane 4). These results indicate that our splicing reporter recapitulates the MeCP2 alternative splicing pattern in Neuro 2a cells. With these results mentioned above, we decided to use Neuro 2a cells for further experiments.

### 2.2. Identification of Cis-Regulatory Element(s) Required for Exon 2 Inclusion

**Figure 2:**
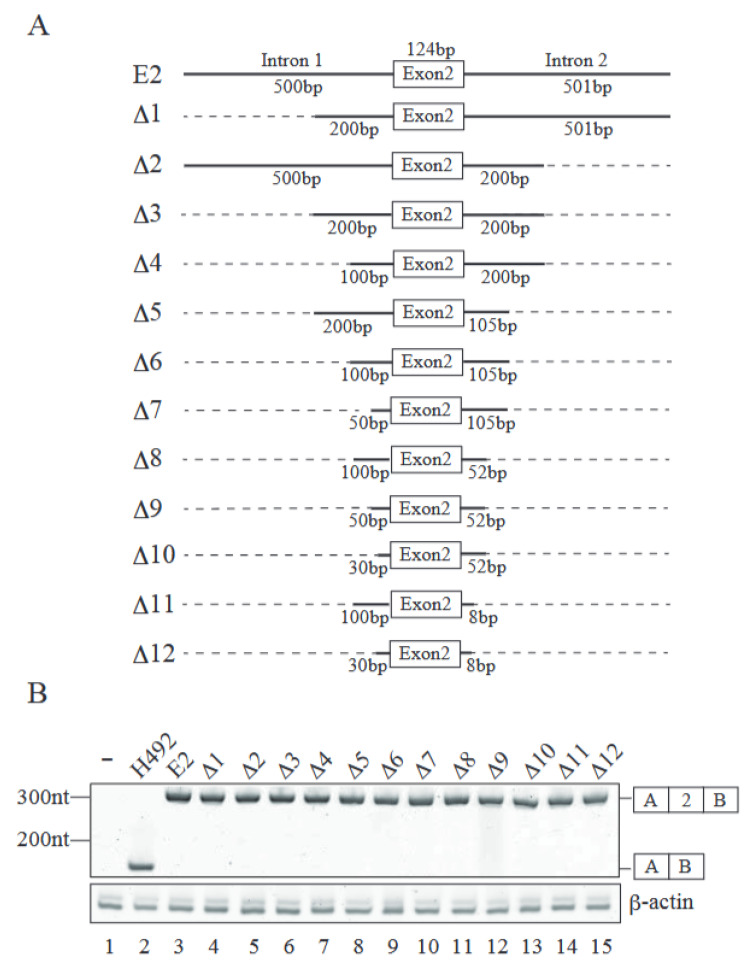
Introns do not contain the element required for exon 2 inclusion. (**A**) Schematic representations of intronic deletion mutant Mecp2 reporters used to identify the element required for exon 2 inclusion. The lengths of residual intronic regions are indicated. Dashed lines indicate the deleted regions, whereas thick lines demonstrate the remaining intron regions. (**B**) The RT-PCR products produced from transfected reporter plasmids were analyzed by acrylamide gel electrophoresis (upper panel). The b-actin mRNA was used as a loading control of RT-PCR reactions (lower panel). The schemes of the RT-PCR products are indicated on the right side of the panel. Representative results from three independent experiments are shown.

**Figure 3:**
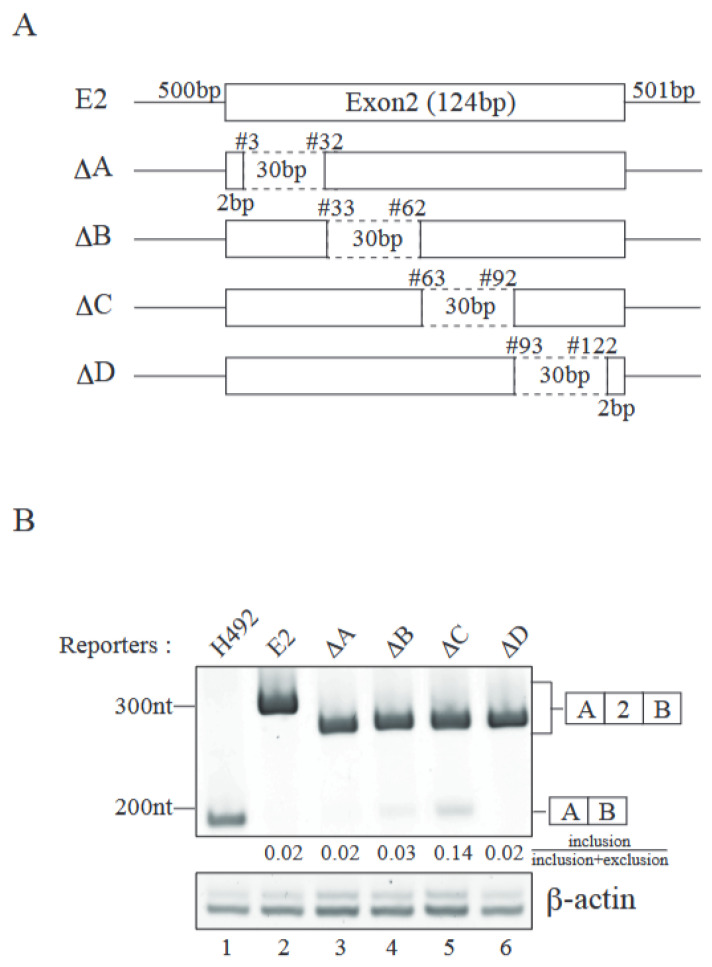
Mecp2 exon 2 contains exonic splicing enhancer(s). (**A**) Schematic representations of exonic deletion splicing reporters. Dashed lines indicate the deleted region of exon 2. The lengths of introns and exon 2 are shown in the E2 reporter. Each deletion reporter has a 30 bp-deletion in a different position of exon 2. The numbers of the nucleotides from the 5′ end of exon 2 are shown above each deleted region. The first and last 2 bp of exon 2 remain intact to avoid the possible effect on splice site recognition. (**B**) The products produced by RT-PCR using RNAs from transfected reporter plasmids were analyzed by acrylamide gel electrophoresis (upper panel). The averages of the ratios of exon 2-included product out of the total mRNA products with two independent experiments are also shown under the panel. The b-actin mRNA was used as a loading control (lower panel). Schematic representations of RT-PCR products are indicated at the right of the panel. Representative results from three independent experiments are shown.

We introduced a series of deletions in both introns 1 and 2 to identify intronic element(s) involved in exon 2 inclusion (Figure 2A). Those deletion mutants were transfected into Neuro 2a cells, and their splicing patterns were analyzed by RT-PCR. To our surprise, all the deletion mutants exhibited only e2-type product (Figure 2B). With these results, it is highly likely that introns 1 and 2 have no intronic elements required for exon 2 inclusion. Then, we attempted to identify the exonic element(s) required for exon 2 inclusion by deleting several regions of exon 2 (Figure 3A). Internal 120 nucleotides of exon 2 were separated into four parts, and each part, comprising 30 nucleotides, was deleted. The resultant plasmids, which were called DA, DB, DC, and DD, were introduced into Neuro 2a cells and total RNAs were recovered. RT-PCR analyses demonstrated that DC reporter, which had a deletion of nucleotides #63–92 of exon 2, delivered the exon 2-excluded e1-type product designated in Figure 3B (lane 5). DB also exhibited a tiny amount of exon 2-excluded product (Figure 3B, lane 4). These results indicated that the nucleotide #63–92 region of *Mecp2* exon 2 harbored the exonic splicing enhancer(s) required for exon 2 inclusion, while #33–62 of exon2 may have had weaker exonic splicing enhancer(s).

### 2.3. Both Splice Sites Adjacent to Exon 2 Contribute to Exon 2 Inclusion

**Figure 4:**
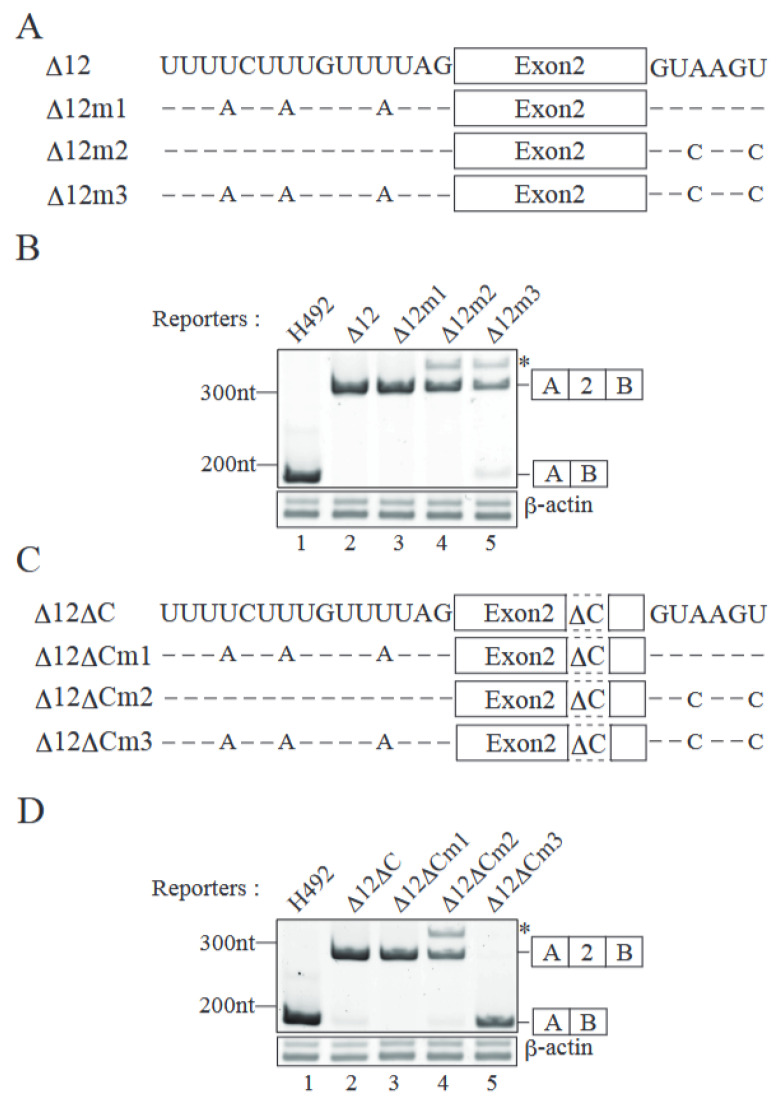
Both splice sites and the ESE of exon 2 mediate exon inclusion in Mecp2 pre-mRNA alternative splicing. (**A**) Mutations in either the polypyrimidine tract or 5′ splice site. A and C designate mutations to adenine and cytosine, respectively. (**B**) RT-PCR analyses of mRNAs generated from the splice site mutant reporters (upper panel). Sequencing analyses reveal that the asterisk indicates the product produced by aberrant splicing utilizing the downstream abnormal 5′ splice site derived from the H492 vector. The β-actin mRNA was employed as an internal loading control (lower panel). The schemes of RT-PCR products are indicated on the right side of the panel. Representative results from three independent experiments are shown. (**C**) Schematic representations of the D12DC reporter plasmids and its derivatives carrying splice site mutations as shown in (**A**). (**D**) Acrylamide gel electrophoresis of RT-PCR products produced from the splicing reporters shown in (**C**). The mRNA of β-actin was used as an internal loading control (lower panel). Schematic representations of RT-PCR products are indicated on the right side of the panel. Representative results from three independent experiments are shown. Sequencing analyses reveal that the asterisk indicates the product produced by aberrant splicing utilizing the downstream abnormal 5′ splice site derived from the H492 vector.

Although deletion of nucleotides #63–92 of exon 2 caused exon 2 exclusion, it was inefficient (Figure 3B, lane 5). Therefore, we next focused on both splice sites of exon 2. The 5′ splice site downstream of exon 2 has a sequence of GUAAGU, which completely matches the mammalian 5′ splice site consensus GURAGU (R is A or G) (Figure 4A, D12). The upstream 3′ splice site is UAG and a preceding long pyrimidine stretch, though it has a single G nucleotide (Figure 4A, D12). By introducing the mutations at both splice sites, we expected to reduce the efficiency of their recognition by splicing factors. In order to check whether the introduced mutations decreased the splice site recognition efficiency or not, we determined the scores of splice sites by the MaxEntScan program [22]. The original score of the 5′ splice site was 10.86, and the substitutions at the 3rd and 6th positions with cytosine reduced the score to 7.16 (Supplemental Table S1). For the 3′ splice site mutations, adenosine substitutions with uracil in the pyrimidine stretch decreased the score from 10.49 to 6.48 (Supplemental Table S1). Taken together, we expected that our mutations at these splice sites would reduce the recognition efficiency. We then introduced a series of mutations as shown in Figure 4A into D12 reporter, which contained the shortest insertion (Figure 2A), producing almost entirely e2-type product (Figure 2B, lane 15 and Figure 4B, lane 2). Although mutations at either the 3′ or 5′ splice site did not cause exon 2 exclusion, mutations at both splice sites produced exon 2-excluded product (Figure 4B lanes 3–5). These results demonstrated that the efficient recognition of exon 2 at both splice sites was required for exon 2 inclusion. However, the efficiency of exon 2 exclusion was not high (Figure 4B, lane 5), suggesting that both ESE and splice sites were involved in exon 2 recognition. We then introduced the splice site mutations in the context of the D12DC reporter, which lacks nucleotides #63–92 of exon 2 and shows a slight exon 2 exclusion pattern (Figure 3B, lane 5 and Figure 4D, lane 2). Mutation in the single splice site did not increase exclusion (Figure 4D, lanes 3 and 4). However, mutations at both splice sites in addition to ESE deletion completely abolished exon 2 inclusion (Figure 4D, lane 5). These results indicate that all the elements (ESE in exon 2 and efficiently recognized splice sites) are required for exon 2 inclusion in *Mecp2* pre-mRNA alternative splicing.

### 2.4. SRSF9 Promotes Exon 2 Inclusion Through Binding to Exon 2 Sequence

**Figure 5:**
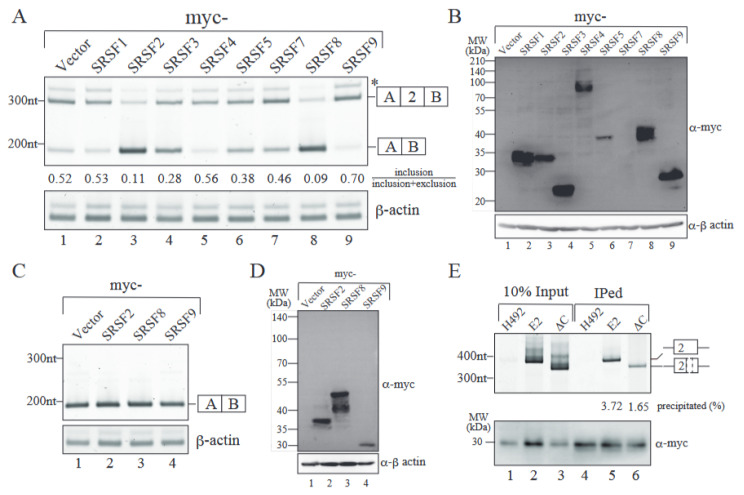
SRSF9 promotes exon 2 inclusion likely through ESE in MeCP2 exon 2. (**A**) Effect of the overexpression of each SR protein in Mecp2 alternative splicing. The RT-PCR products were analyzed by acrylamide gel electrophoresis, followed by quantitation by Image J (version 1.53). The averages of the ratios of the exon 2-included product out of the total mRNA products with two independent experiments are also shown under the panel. The asterisk indicates the product produced by aberrant splicing utilizing the downstream abnormal 5′ splice site, as described in Figure 4B. The β-actin mRNA was used as an internal control. The representative results from three independent experiments are shown. (**B**) The overexpression of each myc-tagged SR protein was analyzed by immunoblotting with anti-myc antibody. The b-actin protein was used as a loading control. The positions of molecular weight size markers are shown at the left of the panel. (**C**) Effect of SR protein overexpression on the alternative splicing of the D12DCm3 splicing reporter. A schematic representation of exon 2-excluded product is indicated on the right. The mRNA of β-actin was used as an internal loading control (lower panel). Representative results from three independent experiments are shown. (**D**) Confirmation of overexpression for exogenously expressed myc-tagged SR protein by immunoblotting with anti-myc antibody. The b-actin protein was used as a loading control. The positions of protein size markers are shown with their molecular weight in kDa. (**E**) RNA immunoprecipitation assay using splicing reporters and myc-SRSF9. (Upper panel) RNAs were co-precipitated with myc-SRSF9 protein by using anti-myc tag antibody. Precipitated RNAs were recovered and analyzed by RT-PCR by using primers hybridizing the intronic region of MeCP2. The percentages of precipitated RNAs are shown. (Lower panel) Precipitated myc-SRSF9 proteins were detected by anti-myc tag antibody. Representative results from three independent experiments are shown.

It has been well accepted that SR proteins bind to ESE and promote exon recognition through interactions with the upstream U2AF complex and the downstream U1 70K protein [2,6]. To test whether SR proteins affect *Mecp2* exon 2 inclusion or not, we co-transfected the plasmids that harbor SR protein cDNAs into Neuro 2a cells along with the D12m3 splicing reporter (Figure 5). With a myc-vector, a negative control plasmid, D12m3 splicing reporter gave us both exon 2-included and -excluded products (Figure 5A, lane 1). The overexpression level of each SR protein could be detected, although the expression level varied (Figure 5B). Among the SRSFs tested, SRSF9 showed the strongest activity in promoting exon 2 inclusion (Figure 5A, lane 9). To our surprise, SRSF2 and 8 had robust activity in enhancing exon 2 exclusion (Figure 5A, lanes 3 and 8). These results indicate that SRSF9 is a candidate to mediate ESE-dependent exon 2 inclusion and that different SR proteins have different modulating activities for MeCP2 alternative splicing. To further test whether SRSF9 promotes exon 2 inclusion through ESE binding or not, we employed the D12DCm3 splicing reporter that has mutations at both splice sites and the deletion of putative ESE (Figure 4C). With this reporter, only exon 2-excluded mRNA was produced (Figure 4D, lane 5 and Figure 5C, lane 1). We then determined the effect of the overexpression of SRSF2, 8, and 9 with this splicing reporter. As shown in Figure 5C, none of the SR proteins tested exhibited exon 2 inclusion (lanes 2–4), although those proteins were comparably expressed (Figure 5D). Finally, we tested whether SRSF9 binds to *MeCP2* exon 2 ESE or not by performing an RNA immunoprecipitation (RIP) assay. Either the E2 reporter or the DC reporter was transfected into Neuro2a cells with the myc-SRSF9 expression plasmid. The H492 vector was used as a negative control. As shown in Figure 5E, myc-SRSF9 precipitated pre-mRNA-containing *MeCP2* exon 2 and the flanking intronic region (Figure 5E, lanes 2 and 5, upper panel). In contrast, the deletion of nucleotides #63–92 of exon 2 (DC) reduced the co-precipitated efficiency with myc-SRSF9 (Figure 5E, lanes 3 and 6, upper panel). Taken together, these results strongly suggest that SRSF9 promotes exon 2 inclusion through binding to ESE in *Mecp2* exon 2.

## 3. Discussion

In this study, we analyzed the alternative splicing mechanism of MeCP2 exon 2 by taking advantage of mini-gene splicing reporters. We first determined the endogenous *Mecp2* pre-mRNA alternative splicing pattern and found that many of the culture cells from humans and mice demonstrated an e2-type dominant splicing pattern, except for mouse TS cells (Figure 1A). Previous studies have demonstrated that *Mecp2* pre-mRNA undergoes alternative splicing in a tissue-specific manner [12,13]. Our splicing reporter exhibited only an exon 2 inclusion pattern in the culture cells we tested (Figure 1A). Even mTS cells only produced exon 2-included mRNA from our splicing reporter, though the endogenous mecp2 gene generated both e1 and e2 isoforms (Figure 1A). We also tested the reporter plasmid that contains the exon1, full-length intron1, exon 2, 1kb of intron 2, and exon3 of *Mecp2*. Unfortunately, this plasmid also gave us only exon 2-included product in both the NIH3T3 and Neuro2a cells (Supplemental Figure S1). It was possible that our splicing reporter did not contain the elements of the *Mecp2* gene required for exon 2 exclusion, likely resigning in a deep region of intron 2. To test this possibility, the whole region of *Mecp2*, expanding from exon 1 to exon 3, should be included in a splicing reporter. However, it is impossible to subclone this region, since the *Mecp2* intron 2 is over 42 kb. To this end, a system different from a plasmid reporter, such as YAC system, should be employed.

Our results indicate that *Mecp2* exon 2 harbors both strong splice sites and ESE. Basically, splicing factors such as U2AF complex and U1 snRNP are efficiently associated with the 3′ and 5′ splice sites adjacent to exon 2, since those splice sites are highly homologous to the splice site consensus sequence. In addition, SRSF9 bound to ESE supports exon recognition for exon 2 by bridging U2AF and U1 snRNP. With the result that our splicing reporters exhibited only the exon 2 inclusion pattern in all the cell lines we tested, it is highly likely that e2-type splicing is a default type for Mecp2 pre-mRNA alternative splicing. In this sense, there should be splicing regulators that cause exon 2 exclusion. SRSF2 and 8 are good candidates for Mecp2 exon 2 exclusion. Since amino acid sequences of the RNA binding domains of SRSF2 and 8 are almost identical, these two proteins are assumed to bind the same RNA sequence. The possible mechanism of how SRSF2 and 8 mediate exon 2 exclusion is that these proteins compete with SRSF9 in binding to ESE in *Mecp2* exon 2. In the nucleotide #63–92 region of *Mecp2* exon 2, there are several purine-rich sequences that SR proteins can associate. Further delineation of the SRSF9 binding site in *Mecp2* exon 2 will shed light on the mechanism for exon 2 inclusion.

The e2 mRNA of *Mecp2* was demonstrated to contain upstream open reading frame (uORF), which reduces the protein expression of the MeCP2E2 protein [12]. Total MeCP2 proteins should be kept at an appropriate protein level in cells, since the duplication of *Mecp2* results in Rett syndrome [9]. Production of e2 mRNA isoforms may contribute to the maintenance of the MeCP2 protein level. Modulation of the *Mecp2* alternative splicing may be able to regulate the protein balance between the MeCP2E1 and MeCP2E2 isoforms. Our results revealed a part of the mechanism for *Mecp2* alternative splicing. Future studies on the complete elucidation of the *Mecp2* alternative splicing mechanism may open up the way to find therapeutic approaches for particular Rett syndrome patients with high expression levels of MeCP2 protein.

## 4. Materials and Methods

### 4.1. Enzymes and Reagents

Restriction enzymes, TransIT-X2 Dynamic Delivery System, and SapphireAmp Fast PCR Master Mix were purchased from Takara Bio, Kusatsu, Japan. The bFGF protein was obtained from WAKO chemicals, Richmond, VA, USA. Activin A and heparin were purchased from R&D Systems, Minnneapolis, MN, USA and Sigma-Aldrich, Tokyo, Japan, respectively. RQ1 RNase-Free DNase and ReverTra Ace qPCR RT Kit were obtained from Promega, Madison, WI, USA and TOYOBO, Novi, MI, USA, respectively.

### 4.2. Plasmid Construction

The H492 vector was a kind gift from Dr. Masafumi Matsuo (Kobe Tokiwa University) [23]. To construct the pH492-E2 reporter plasmid, the mouse *Mecp2* genomic DNA fragment encompassing exon 2 and flanking intronic regions was amplified by PCR using E2-F and E2-R primers (Table 1). The amplified product was cloned between the NheI and BamHI sites of the H492 vector. The pH492-E2 plasmid was used as a template in the series of intron deletion mutants (∆1–∆12). A series of exon deletion mutants (∆A–∆D) were amplified by inverse PCR. The splice site mutation plasmids (∆12m1–m3 and ∆12∆Cm1–m3) were prepared using the ∆12 plasmid or ∆C plasmid as a template for PCR. The primers used for these PCRs are shown in Table 1.

The plasmids expressing myc-tagged SRSF proteins were described previously [24].

### 4.3. Cell Culture and Transfection

NIH3T3 cells were cultured at 37 °C with 5% CO_2_ in DMEM (High Glucose) supplemented with 10% (*v*/*v*) fetal bovine serum (FBS), 100 units/mL penicillin, and 100 µg/mL streptomycin. Neuro 2a cells were maintained in EMEM supplemented with 10% FBS, 1% MEM Non-essential Amino Acids Solution (Wako Pure Chemical Industries, Osaka, Japan), 100 units/mL penicillin, and 100 µg/mL streptomycin at 37 °C with 5% CO_2_. C57BL/6 mouse Trophoblast Stem Cells (mTSCs) (B6TS2) established in our laboratory were used in this study [25]. To maintain an undifferentiated state, the cells were cultured in TS cell medium [21] supplemented with 10 ng/mL activin A, 1 µg/mL heparin, and 25 ng/mL basic fibroblast growth factor. Transfection was carried out by using polyethylenimine (PEI) and TransIT-X2 Dynamic Delivery System according to the manufacturer’s recommendation and as described previously [26].

### 4.4. RNA Preparation and Reverse Transcription Polymerase Chain Reaction (RT-PCR)

Total RNA preparation from transfected cells was performed by using TRI Reagent (Molecular Research Center, Cincinnati, OH, USA) according to the manufacturer’s recommendation after 24 h of transfection. A total of 0.5 µg of total RNA was treated with RQ1 RNase-Free DNase and subjected to cDNA synthesis using ReverTra Ace qPCR RT Kit (TOYOBO). The produced cDNA was used for PCR using SapphireAmp Fast PCR Master Mix (Takara Bio.). The cycle conditions were as follows: 94 °C for 1 min, followed by 28 cycles (*Mecp2* and *Actb*) or 35 cycles (reporters) of 98 °C denaturation for 5 s; 60 °C (*Mecp2* and *Actb*) or 56 °C (reporters) annealing for 5 s; and 72 °C elongation for 5s, with a final incubation at 72 °C for 2 min in a PCR Thermal Cycler (BIOMETRA, Gottingen, Germany). PCR products were separated by acrylamide gel electrophoresis and stained with Midori Green Xtra (NIPPON GENE, Toyama, Japan). The primers for RT-PCR and RT-qPCR are shown in Table 1.

### 4.5. Antibodies and Immunoblotting Analyses

Transfected cells were suspended with sample buffer solution with the reducing reagent for SDS-PAGE (Nacalai Tesque, Kyoto, Japan) and boiled for 5 min. The samples were resolved on 12.5% SDS PAGE and transferred to an Immobilon PVDF membrane (Millipore, Burlington, MA, USA) by electroblotting. The membranes were blocked with 5% skimmed milk in TBS containing 0.1% Tween 20 for 1 h at room temperature and probed for 1 h at room temperature with c-Myc antibody (1:500 dilution; 9E10, sc-40, Santa Cruz Biotechnology, Dallas, TX, USA) or anti-β-actin mAb (1:2000 dilution; Medical & Biological Laboratories, Fukuoka, Japan). The bound antibodies were detected with peroxidase-conjugated goat anti-mouse IgG antibodies (Jackson ImmunoResearch Laboratories, West Grove, PA, USA) and signals were developed with Chemi-Lumi One Super detection reagents (Nacalai Tesque, Kyoto, Japan). The signals were detected by GeneGnome System High Resolution 100 (SYNGENE, Cambridge, UK).

### 4.6. Calculation of the Splice Site Scores by MaxEntScan Program

The scores for both wild type and mutated splice site sequences shown in the SupplementalTable S1 were calculated by the MaxEntScan program accessed on 29 March 2024 (for 5′ splice site, http://hollywood.mit.edu/burgelab/maxent/Xmaxentscan_scoreseq.html, for 3′ splice site, http://hollywood.mit.edu/burgelab/maxent/Xmaxentscan_scoreseq_acc.html) [22].

### 4.7. RNA Immunoprecipitation (RIP) Assay

Neuro 2a cells grown in a 6 cm dish were transfected with the myc-SRSF9 plasmid and mini-gene reporter. Transfected cells were harvested in 200 µL NET2 buffer (150 mM NaCl, 0.05% NP-40, 50 mM Tris-HCl [pH 7.5]). Cell lysates were sonicated using a VP-15S Ultras Homogenizer (TAITEC, Koshigaya, Japan) and centrifuged for 20 min at 15,000× *g* at 4 °C. The supernatant was incubated with 16 mL of Anti-c-Myc-Agarose (Nacalai Tesque, Kyoto, Japan) in the presence of 1U/µL Recombinant RNase Inhibitor (Takara Bio) for 1 h at 4 °C with gentle rotation. The beads were placed on the spin column (Aji Bio-Pharma, San Diego, CA, USA) and centrifuged for 30 s at 8000× *g*. The flowthrough was discarded, and the beads were washed with 500 µL NET2 buffer. This step was repeated five times. The beads were resuspended in 400 µL NET2 and divided into two aliquots (for WB and RT-PCR). Immunoprecipitated RNA and input RNA were isolated by using TRI Reagent (Molecular Research Center) and treated with RQ1 RNase-Free DNase (Promega, Madison, WI, USA) to remove contaminated DNAs. Reverse transcription was performed by using PrimeScript Reverse Transcriptase (Takara Bio) with reporter-specific SP6 promoter primer. RT-PCR for Mecp2 pre-mRNA was performed with the specific primers described in Table 1 by using SapphireAmp Fast PCR Master Mix (Takara Bio). PCR products were analyzed by acrylamide gel electrophoresis. Proteins were isolated by the sample buffer and detected by immunoblotting analysis.

## Figures and Tables

**Table 1:** A list of the primers used for plasmid construction and RT-PCR.

**Plasmid Construction**		
**Plasmid**	**Primer Name**	**Primer (5′-3′)**
E2	F	AAAGCTAGCGAAGCAGAAGAGAGACTTTTGCCT
	R	AAAGGATCCCTCCATTGTGCCGTGACACTTTCA
∆1	F	AAAGCTAGCTTCTGACAGCCATAACTCTTGTCT
	R	AAAGGATCCCTCCATTGTGCCGTGACACTTTCA
∆2	F	AAAGCTAGCGAAGCAGAAGAGAGACTTTTGCCT
	R	AAAGGATCCAAACTGAACTGAACTTTCAGGGCT
∆3	F	AAAGCTAGCTTCTGACAGCCATAACTCTTGTCT
	R	AAAGGATCCAAACTGAACTGAACTTTCAGGGCT
∆4	F	AAAGCTAGCACATAGTATGTTGTCTGTTTATC
	R	AAAGGATCCAAACTGAACTGAACTTTCAGGGCT
∆5	F	AAAGCTAGCTTCTGACAGCCATAACTCTTGTCT
	R	AAAGGATCCCATACTGTCCAAGAATAGTATATC
∆6	F	AAAGCTAGCACATAGTATGTTGTCTGTTTATC
	R	AAAGGATCCCATACTGTCCAAGAATAGTATATC
∆7	F	AAAGCTAGCAAAGGTGCAGCTCAATGGGG
	R	AAAGGATCCCATACTGTCCAAGAATAGTATATC
∆8	F	AAAGCTAGCACATAGTATGTTGTCTGTTTATC
	R	AAAGGATCCAGATGGCCAAACCAGGACATATAC
∆9	F	AAAGCTAGCAAAGGTGCAGCTCAATGGGG
	R	AAAGGATCCAGATGGCCAAACCAGGACATATAC
∆10	F	AAAGCTAGCACTTTCAACTTACGATTTTCTTTG
	R	AAAGGATCCAGATGGCCAAACCAGGACATATAC
∆11	F	AAGCTAGCAAAGGTGCAGCTCAATGGGG
	R	AAAGGATCCTTACTTACCTGAGCCCTAACATC
∆12	F	AAAGCTAGCACTTTCAACTTACGATTTTCTTTG
	R	AAAGGATCCTTACTTACCTGAGCCCTAACATC
∆A	F	GCCTAAAACAAAGAAAATCGTAAGTTG
	R	CTTTGATGTGACATGTGACTCCC
∆B	F	CAGGAACTGGTGAGTCTGTATTTTTATG
	R	CACCTTGCTTCTGTAGACCAGCTC
∆C	F	TATTCTGGGGAGTCACATGTCACATC
	R	GATTCCATGGTAGCTGGGATGTTAG
∆D	F	CTGTTGGAGCTGGTCTACAGAAG
	R	AGGTAAGTAACCTTCCTTTTTTTTTT
∆12m1 ∆12∆Cm1	F	AAAGCTAGCACTTTCAACTTACGATTTACTATGTTATAG
	R	AAAGGATCCTTACTTACCTGAGCCCTAACATC
∆12m2 ∆12∆Cm2	F	AAAGCTAGCACTTTCAACTTACGATTTTCTTTG
	R	AAAGGATCCTTGCTGACCTGAGCCCTAAC
∆12m3 ∆12∆Cm3	F	AAAGCTAGCACTTTCAACTTACGATTTACTATGTTATAG
	R	AAAGGATCCTTGCTGACCTGAGCCCTAAC
**RT-PCR**		
**Target**	**Primer Name**	**Primer Sequence (5′-3′)**
Mecp2	F	GGTAAAACCCGTCCGGAAAATG
	R	TTCAGTGGCTTGTCTCTGAG
Actb	F	TTCTACAATGAGCTGCGTGTGG
	R	ATGGCTGGGGTGTTGAAGGT
Reporters	F	ATTACTCGCTCAGAAGCTGTGTTGC
	R	AAGTCTCTCACTTAGCAACTGGCAG
SP6 promoter	SP6 promoter primer	GATTTAGGTGACACTATAG
MeCP2 pre-mRNA	F	AAAGCTAGCACATAGTATGTTGTCTGTTTATC
MeCP2 pre-mRNA	R	AAAGGATCCCATACTGTCCAAGAATAGTATATC

## Data Availability

The original contributions presented in this study are included in the article/Supplementary Material. Further inquiries can be directed to the corresponding author.

## Supplementary Materials

The supporting information can be downloaded at: https://www.mdpi.com/article/10.3390/ijms26073319/s1.
