# Supplementary material for: SRSF9-Mediated Exon Recognition Promotes Exon 2 Inclusion in Mecp2 Pre-mRNA Alternative Splicing
Source: Int J Mol Sci. 2025 Apr 2;26(7):3319. doi: 10.3390/ijms26073319 (PMC11989674; doi:10.3390/ijms26073319)
Supplement: Supplementary file 1 [file ijms-26-03319-s001.zip › ijms-3554411-supplementary.pdf]

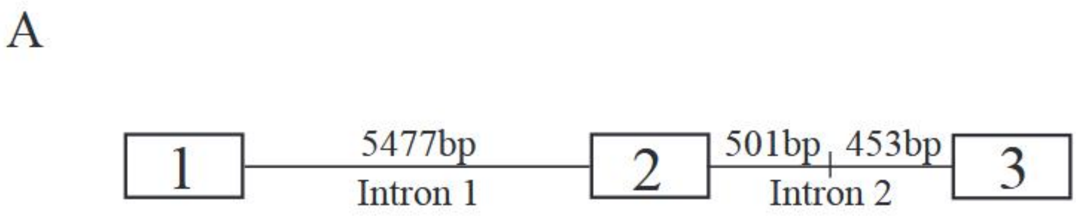

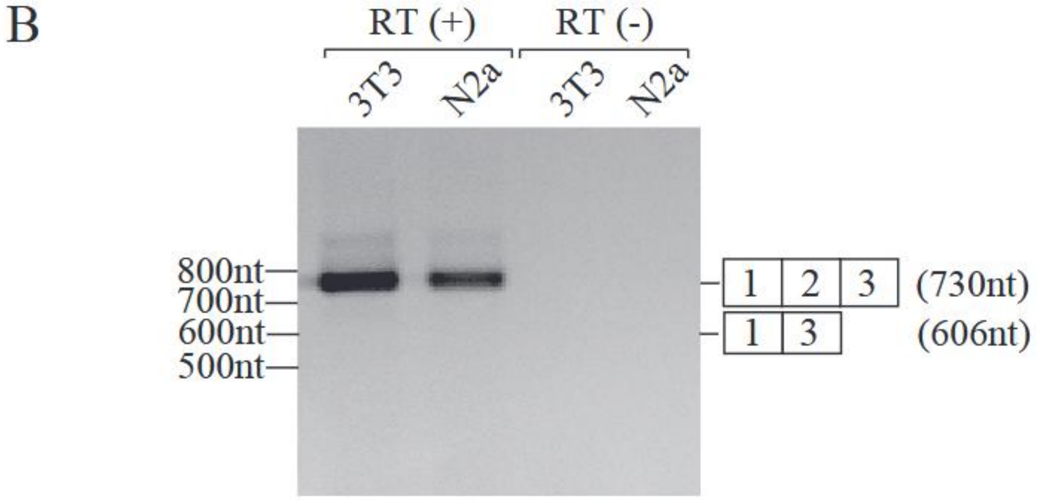

Supplemental Figure 1. A mini-gene splicing reporter carrying Mecp2 exons 1-3, full length of intron 1 and a portion of intron 2. (A) A schematic representation of *Mecp2* region inserted into pCDNA3 plasmid. (B) Splicing patterns of the splicing reporter described in (A) in NIH3T3 (3T3) and Neuro2a (N2a) cells. Representative results from three independent experiments are shown.

Supplemental Table 1. MaxEntScan scores for wild type and mutated splice sites

	MaxEnt Score for 5' splice site	MaxEnt Score for 3' splice site	Sequence of 5' splice site	Sequence of 3' splice site
WT	10.86	10.49	cagGTAAGT	tacgattttctttgttttAGgct
m1	10.86	6.48	cagGTCAGC	tacgattttctttgttttAGgct
m2	7.16	10.49	cagGTAAGT	tacgatttactatgttatAGgct
m3	7.16	6.48	cagGTCAGC	tacgatttactatgttatAGgct
